# Incremental Prognostic Value of the C-Reactive Protein-to-Albumin Ratio Beyond a Parsimonious Clinical Reference Model in Critically Ill Patients with Acute Ischemic Stroke

**DOI:** 10.3390/diagnostics16162578

**Published:** 2026-08-15

**Authors:** Hasan Burak Toprak, Mete Erdemir, Elif Bilgiç, Cevdet Furkan Köşker, Şerife Bozdaş, Meltem Bilge, Gürhan Taşkın, Levent Yamanel

**Affiliations:** Department of Intensive Care, Gulhane Training and Research Hospital, Gen. Dr. Tevfik Sağlam Street No:1, Keçiören 06010, Ankara, Türkiye; meteerdemir@hotmail.com (M.E.); dr.elifbilgic@gmail.com (E.B.); furkankskr@gmail.com (C.F.K.); serifecetinkaya_756@hotmail.com (Ş.B.); doktormeltem@hotmail.com (M.B.); drgurhantaskin@gmail.com (G.T.); lyamanel@gmail.com (L.Y.)

**Keywords:** acute ischemic stroke, C-reactive protein-to-albumin ratio, prognosis, incremental value, risk prediction, intensive care units

## Abstract

**Background and Objectives:** The C-reactive protein-to-albumin ratio (CAR) is associated with mortality and poor outcome after acute ischemic stroke, but the association is not the same as added clinical usefulness. We evaluated whether admission CAR and follow-up CAR provide prognostic information beyond a prespecified parsimonious clinical reference model comprising age, neurological severity, and admission glucose in critically ill patients with acute ischemic stroke. **Materials and Methods:** In this single-center retrospective cohort of 146 adults with acute ischemic stroke managed in intensive care, CAR was calculated from C-reactive protein and serum albumin at emergency department admission and at the first intensive care laboratory assessment. The primary outcome was 90-day all-cause mortality. A clinical reference model (age, admission National Institutes of Health Stroke Scale [NIHSS] score, admission glucose) was compared with the same model augmented by log-transformed CAR using the area under the receiver operating characteristic curve (AUC), the DeLong test, likelihood-ratio (LR) testing, and bootstrap optimism-corrected performance. **Results:** Ninety-day mortality occurred in 32 of 146 patients (21.9%) (full cohort; primary complete-case analysis: 141 patients with 31 events). In the primary complete-case analysis (*n* = 141; 31 events), admission CAR was not independently associated with mortality (odds ratio per 1-SD log CAR 1.46, 95% confidence interval 0.91–2.33; *p* = 0.115). Adding admission CAR changed the AUC from 0.768 (0.676–0.860) to 0.783 (0.693–0.874) (ΔAUC +0.016, 95% confidence interval −0.024 to +0.055; DeLong *p* = 0.444; LR *p* = 0.113), with optimism-corrected point estimates of 0.750 and 0.754. Follow-up CAR did not add value (LR *p* = 0.159), and change in CAR did not improve discrimination (ΔAUC +0.001; LR *p* = 0.945); because intensive care sampling times were not standardized, these analyses assess incremental prognostic information rather than CAR kinetics. Admission CAR was not associated with poor 90-day functional outcome (odds ratio 1.09, 95% confidence interval 0.83–1.44; *p* = 0.530). **Conclusions:** Admission CAR did not demonstrate measurable incremental prognostic value beyond the prespecified clinical reference model, and follow-up CAR and change-based analyses did not improve prediction, although non-standardized sampling times mean that serial CAR kinetics were not fully evaluated. The confidence intervals remain compatible with both no effect and a positive effect of uncertain clinical relevance, but the observed improvement was insufficient to support CAR as a stand-alone or routinely additive prognostic marker in this setting.

## 1. Introduction

Systemic inflammation is closely linked to outcomes after acute and critical illness. C-reactive protein (CRP) reflects acute-phase inflammatory activation, whereas serum albumin integrates nutritional reserve, hepatic synthetic capacity, and chronic inflammatory burden. The C-reactive protein-to-albumin ratio (CAR) combines these complementary signals and has therefore been proposed as a practical composite inflammatory marker [1].

Initial evidence supporting CAR came from oncological and general critical care populations, where higher CAR was associated with postoperative or short-term mortality [1,2,3,4]. Subsequent studies extended this association to heterogeneous intensive care, sepsis, acute kidney injury, severe burns, acute pancreatitis, coronavirus disease 2019, and other systemic inflammatory states [5,6,7,8,9,10,11,12]. Further studies evaluated CAR in relation to post-acute COVID-19 pulmonary function, cardiovascular and neurological disease severity or prognosis, and mortality in sepsis, acute kidney injury, and adult or pediatric intensive care cohorts [13,14,15,16,17,18,19,20,21,22]. Collectively, this literature positioned CAR as a marker associated with disease burden and adverse outcomes, while leaving uncertain whether it improves prognostication after established clinical predictors are considered.

Prior work in acute ischemic stroke has repeatedly linked a high CAR to worse prognosis. Kocatürk and Kocatürk reported that CAR was an independent predictor of 90-day mortality [23]. Related studies associated CAR with adverse outcomes after traumatic brain injury or intracerebral hemorrhage and examined its relationship with etiology and prognosis in acute ischemic stroke [24,25,26]. A recent meta-analysis pooled multiple cohorts and found that elevated CAR was associated with both mortality and poor functional outcome [27]. Additional stroke-focused studies reported associations with hemorrhagic transformation, functional outcome, mortality, adverse prognosis, or nutritional and sarcopenia-related status across diverse acute-stroke and population-based cohorts [28,29,30,31,32,33]. Yet these reports primarily quantified association, not added value, leaving open the question that determines clinical utility: whether CAR adds prognostic information once the strongest routine predictors—the National Institutes of Health Stroke Scale (NIHSS) score, a deficit scale whose inter-rater reliability supports its routine use [34], age, and admission glucose—are already in the model. Most analyses reported the association of CAR with outcome rather than its incremental value over a clinical reference model, and few examined whether a follow-up-CAR measurement, obtained under non-standardized intensive care sampling times, improves prediction. Association and incremental usefulness are distinct properties, and a marker can be associated with outcome yet add nothing measurable to an existing model.

We therefore evaluated, in a cohort restricted to critically ill adults with acute ischemic stroke, whether admission CAR, follow-up (intensive care) CAR, or change in CAR adds prognostic value beyond a parsimonious clinical model comprising age, admission NIHSS score, and admission glucose. We assessed discrimination with paired AUC comparison and likelihood-ratio testing, internal validity with bootstrap optimism correction, and the dependence of any signal on the choice of C-reactive protein detection limit and on the timing of follow-up sampling.

## 2. Materials and Methods

### 2.1. Study Design and Population

**Figure 1:**
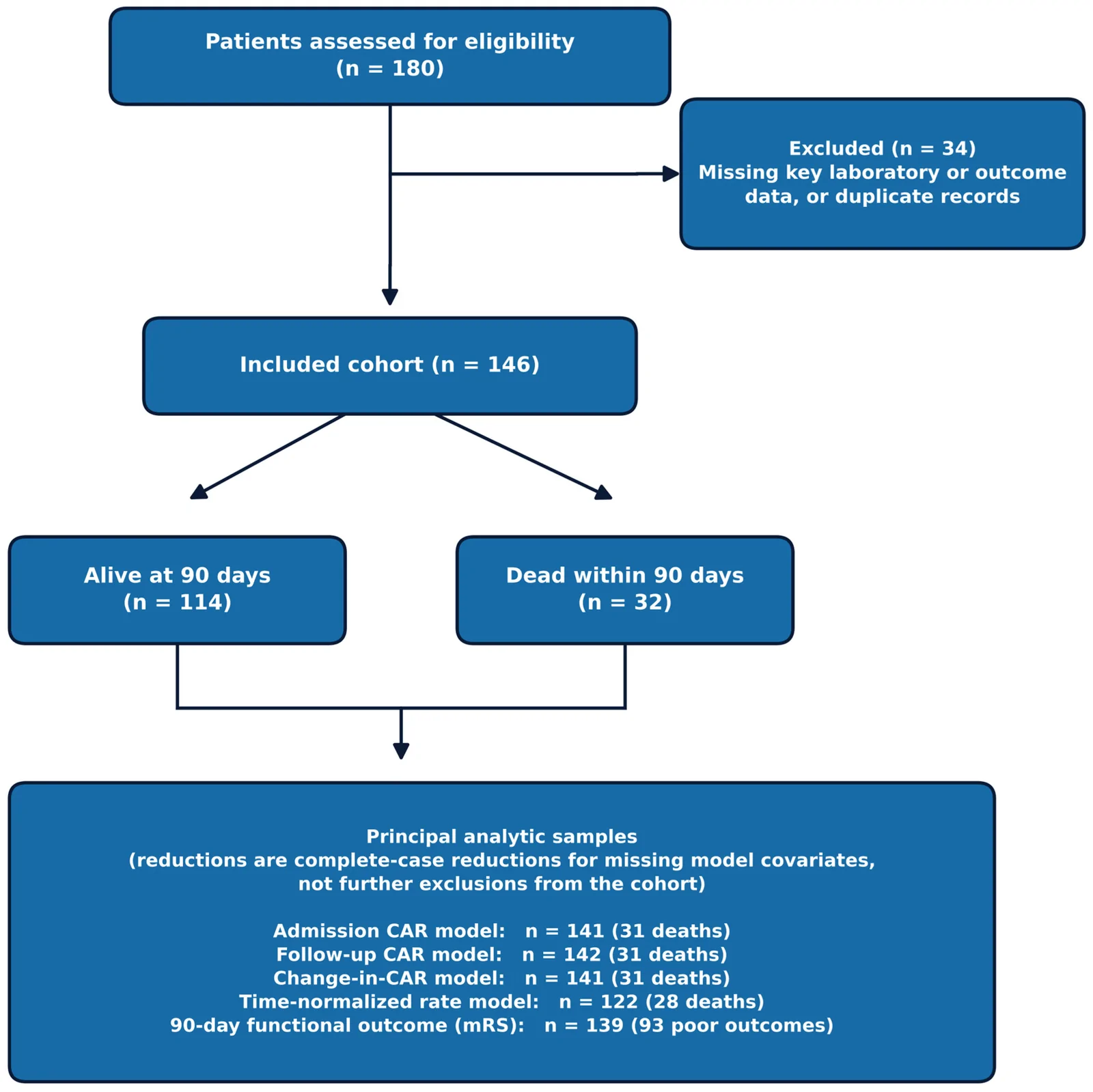
Patient selection and study flow diagram. The diagram represents the screening of consecutive patients and exclusions during cohort assembly, yielding the final cohort of 146 critically ill patients with acute ischemic stroke. Of 180 patients assessed for eligibility, 34 were excluded because of missing key laboratory or outcome data, or duplicate records; a per-category breakdown was not retained in the screening records. This left an included cohort of 146, of whom 114 were alive, and 32 had died at 90 days. The final panel gives the complete-case sample size and event count of the principal analytic samples: admission CAR *n* = 141 (31 deaths), follow-up CAR *n* = 142 (31 deaths), change in CAR *n* = 141 (31 deaths), time-normalized rate of change *n* = 122 (28 deaths), and 90-day functional outcome *n* = 139 (93 patients with a modified Rankin Scale score of 3 to 6). These reductions are complete-case reductions caused by missing model covariates and are not further exclusions from the cohort.

This retrospective observational cohort study was conducted in adult patients admitted through the emergency department with acute ischemic stroke and subsequently managed in an intensive care unit. Consecutive patients treated between January 2020 and December 2024 were screened. Eligibility required age 18 years or older, a clinical and radiological diagnosis of acute ischemic stroke, intensive care management, and availability of the primary outcome and baseline clinical data. Patients with missing outcome data, incomplete clinical records, or duplicate records were excluded. Predictor-specific analyses were subsequently conducted using complete cases for the variables required by each model. The patient selection process is shown in Figure 1. The study followed the Strengthening the Reporting of Observational Studies in Epidemiology framework.

The cohort should therefore be interpreted as a critically ill acute ischemic stroke cohort rather than as a general all-comer acute ischemic stroke population.

### 2.2. Variables and Definitions

Demographic variables included age and sex. Clinical variables included admission NIHSS score, Glasgow Coma Scale score, comorbidities, admission blood glucose, intravenous thrombolysis, mechanical thrombectomy, reperfusion status where available, intensive care length of stay, intubation, vasopressor requirement, aspiration pneumonia, intracranial hemorrhage, cerebral edema, and sepsis recorded during hospitalization or intensive care follow-up.

CRP was measured by immunoturbidimetry and serum albumin by a colorimetric method on a Beckman Coulter AU5800 analyzer (Beckman Coulter, Brea, CA, USA) at the hospital central laboratory. CAR was calculated as CRP divided by serum albumin. CRP values were recorded in mg/L and albumin values in g/dL; therefore, CAR values are reported as mg/L per g/dL. This unit convention is stated explicitly because it is not universal. A CRP of 40 mg/L with a serum albumin of 3.2 g/dL gives a CAR of 12.5 mg/L per g/dL, whereas a study expressing both analytes in the same mass units would report a numerically different value for the same patient. No unit conversion was applied here, so absolute CAR values in this paper should not be compared directly with values from studies using a different convention. The admission and follow-up CAR analyses are invariant to a constant unit conversion, because CAR was modeled on the log scale and reported per standard deviation. For the raw change-in-CAR and time-normalized rate analyses, which are modeled on the untransformed scale, a unit conversion would rescale the regression coefficient, although it would not change the fitted probabilities, the likelihood-ratio tests, or discrimination. CAR was calculated from time-matched measurements: admission CAR from admission CRP and admission serum albumin, and intensive care CAR from the corresponding intensive care values. Change in CAR therefore reflects a genuine within-patient change between the two measurement time points. C-reactive protein values recorded as 0 mg/L were interpreted as below the assay reporting limit rather than true zeros and were imputed as the detection limit divided by the square root of two (DL/√2), with DL set to 1 mg/L (integer reporting; reference interval 0–10 mg/L); the robustness of the primary incremental analysis to this assumption was examined across detection limits of 1, 3, and 5 mg/L. CAR was highly right-skewed and was therefore analyzed as log(CAR) in regression models, and the primary cohort of 146 patients was preserved, with complete-case reductions arising only from missing covariates. Delta CAR was calculated as intensive care CAR minus admission CAR and was analyzed on its raw scale.

The intensive care follow-up CAR measurement corresponded to the first available intensive care laboratory assessment recorded in the source dataset. The interval between emergency department and intensive care laboratory sampling had a median of 8.7 h (interquartile range 6.3–12.9), with 94.4% of intensive care samples obtained within 24 h and 99.2% within 48 h of the emergency department sample; an interval was treated as evaluable only if it fell inside the predefined analytic window of more than 0 h and at most 168 h (7 days), so that negative intervals, intervals of exactly zero hours (both samples carrying the same recorded timestamp), and intervals exceeding 168 h were set to missing for this calculation. A valid interval was available for 125 patients, and this rule was applied identically to every interval-dependent analysis reported below. Because follow-up sampling times were not standardized across all patients, follow-up CAR analyses were interpreted cautiously and were used to test incremental prognostic information rather than time-normalized biomarker change.

Sepsis was coded from the source dataset as a binary documented hospitalization or intensive care complication. Uniform retrospective reclassification according to Sepsis-3 criteria and SOFA score was not possible from the available dataset. To avoid unsupported causal interpretation and overadjustment, sepsis was not included in the primary baseline prognostic model; instead, it was evaluated in a separate clinical-course sensitivity model.

### 2.3. Outcomes

The primary outcome was 90-day all-cause mortality, recorded in the source clinical follow-up dataset. Patients with missing 90-day outcome data were excluded from the analytic cohort. Outcomes were ascertained from the clinical follow-up record independently of laboratory predictor values. Ninety-day vital status and modified Rankin Scale score were ascertained exclusively from hospital electronic medical records and institutional follow-up documentation; no telephone or other direct contact with patients or relatives was undertaken for this study.

A secondary outcome was poor functional outcome at 90 days, defined as a modified Rankin Scale score of 3 to 6. The modified Rankin Scale analysis was included to improve clinical relevance beyond mortality alone.

### 2.4. Statistical Analysis

Continuous variables were summarized as median with interquartile range, and categorical variables were summarized as counts and percentages. Between-group comparisons according to 90-day mortality used the Mann–Whitney U test for continuous variables and chi-square or Fisher exact testing for categorical variables, as appropriate.

Given the limited number of mortality events, the primary multivariable analysis was intentionally parsimonious. With 31 events and four parameters in the primary model, the events-per-variable ratio was approximately 7.8, below the conventional threshold of 10; model size was therefore constrained a priori and no data-driven predictor selection was performed. The clinical reference model included age, admission NIHSS score, and admission glucose. These three variables were selected on clinical grounds because they are routinely available at emergency department presentations and because they represent three distinct and dominant prognostic axes after acute ischemic stroke: chronological age, neurological severity, and acute metabolic stress. Glasgow Coma Scale score was not included in the primary model because it is substantially correlated with the admission NIHSS score in this population (Pearson r = −0.69; variance inflation factor 2.02 when the two are entered together) and therefore restates the axis of neurological severity that the NIHSS score already represents. The correlation is not strong enough to destabilize joint estimation, so this is a decision about parsimony and events per variable rather than about collinearity: with 31 events, retaining Glasgow Coma Scale score would have consumed a further degree of freedom without introducing an independent prognostic axis. A reference model that includes it is reported as a sensitivity analysis in the Supplementary Materials. Reperfusion treatment, infarct characteristics, premorbid modified Rankin Scale score, and stroke etiology were likewise excluded from the primary model for two reasons. First, several of these variables, specifically infarct volume, premorbid modified Rankin Scale score, and TOAST subtype, were not uniformly recorded in the source dataset and could not be reconstructed retrospectively. Second, with 31 events, any expansion of the model beyond four parameters would move the events-per-variable ratio further below the conventional threshold and would increase overfitting rather than reduce confounding. The direction in which this choice acts should be stated carefully: because the question is whether CAR adds information beyond an existing clinical model, a deliberately small reference model gives an incremental contribution the best opportunity to appear. Among the expanded models that could be fitted, the parsimonious primary model was empirically the configuration most favorable to CAR; the direction and magnitude of any bias arising from variables that were not recorded cannot be determined from these data. Published multivariable stroke prognostic scores such as ASTRAL [35], iScore [36], and PLAN [37] were not used as the reference model, because they require variables that were not uniformly available in this dataset and because the aim was to test the incremental value of a single laboratory marker rather than to propose, benchmark, or validate a prognostic score. Sensitivity analyses in which the reference model was expanded with Glasgow Coma Scale score, receipt of reperfusion treatment, and time from symptom onset are reported in the Supplementary Materials. All three clinical predictors were prespecified rather than selected by data-driven procedures, and the log transformation of CAR was specified before modeling; LASSO was used only as an exploratory check. Admission CAR was then added to this model to evaluate independent association and incremental prognostic value. Follow-up analyses separately evaluated intensive care CAR and change in CAR (an exploratory analysis), added to the same clinical reference structure. Model performance was summarized using area under the receiver operating characteristic curve, Brier score, Akaike information criterion, and likelihood-ratio testing for nested comparisons. Calibration was assessed with a locally weighted scatterplot smoother (loess). Internal validity was assessed by bootstrap resampling (1000 resamples), reporting optimism-corrected AUC, Brier score, and calibration slope and intercept. Because an optimism-corrected AUC is itself an estimate, a confidence interval was obtained for it by a nested bootstrap: in each of 600 outer resamples, the optimism correction was repeated with an inner bootstrap of 120 resamples, and the 2.5th and 97.5th percentiles of the resulting distribution are reported as the interval. Missing data were handled by complete-case analysis for each model. Missingness in model variables ranged from 0% to 2.1% (admission glucose); per-variable counts are reported in Supplementary Table S1. In total, 5 of 146 patients (3.4%) had at least one missing model variable. Missingness was assumed to be at random conditional on the observed data. This assumption cannot be verified, and it is not testable in these data. The observed pattern is at least not inconsistent with it: the proportion of deaths did not differ detectably between patients with and without a missing value for admission NIHSS score (0 of 1 versus 32 of 145; Fisher exact *p* = 1.000), admission glucose (1 of 3 versus 31 of 143; *p* = 0.527), or emergency department C-reactive protein (0 of 2 versus 32 of 144; *p* = 1.000). With only one to three missing values per variable, these comparisons have almost no power and therefore cannot be treated as evidence in favor of the assumption; they establish only that no gross outcome-related pattern is visible. Each missing value corresponded to an absent routine laboratory or scoring entry in the source record; with these numbers, outcome-related missingness cannot be excluded. Complete-case analysis was preferred because it does not require an imputation model to be specified and fitted in a sample containing 31 events. Multiple imputation was not used given the small event count and the analytic rather than deployment aim.

Secondary and sensitivity analyses evaluated poor 90-day functional outcome, clinical-course adjustment including sepsis during hospitalization or intensive care, interaction between admission CAR and NIHSS, and NIHSS-stratified CAR associations. Net reclassification improvement, integrated discrimination improvement, and LASSO-based penalized regression were computed as exploratory analyses; because these estimates are unstable in a 146-patient cohort with 32 deaths, they were interpreted with caution rather than as primary evidence. Individual analyses report nominal, unadjusted *p*-values, and no adjustment for multiple comparisons was applied to any individual test; Holm-adjusted and Benjamini–Hochberg-adjusted values across the full family of incremental-value tests are reported in the Supplementary Materials as a multiplicity sensitivity summary rather than as the inferential basis for any single comparison. The primary analysis, the incremental value of admission CAR over the prespecified clinical reference model, was specified in advance and constitutes a single confirmatory test; every other analysis reported here is secondary or exploratory. Because those analyses are numerous and unadjusted, multiplicity inflates the risk of type I error among them, and no individual secondary *p*-value close to 0.05 should be read as evidence of an effect. The consequences of this for the present results are quantified in the Discussion and in the Supplementary Materials. Two-sided *p*-values less than 0.05 were considered statistically significant.

## 3. Results

### 3.1. Study Population

A total of 180 patients were screened, and 146 critically ill adults with acute ischemic stroke were included after exclusion of 34 patients with missing key data, incomplete outcome information, or duplicate records (Figure 1). Ninety-day mortality occurred in 32 patients (21.9%) of the full cohort of 146. The primary complete-case model included 141 patients with 31 events; the difference of five patients and one event reflects missing model covariates rather than any further exclusion, and the complete-case sample size of each principal analytic sample is shown in Figure 1. Poor 90-day functional outcome was present in 95 of 144 patients with available modified Rankin Scale data (66.0%).

Baseline characteristics are shown in Table 1. Patients who died by 90 days were older, had higher admission NIHSS scores, lower admission Glasgow Coma Scale scores, higher admission glucose levels, longer intensive care stays, and more frequent intubation, vasopressor requirement, sepsis, intracranial hemorrhage, and cerebral edema. Admission CAR was numerically higher among non-survivors but did not differ significantly in unadjusted group comparison.

### 3.2. Admission CAR and 90-Day Mortality

In the primary complete-case model (*n* = 141; 31 events), age, admission NIHSS score, and admission glucose were each independently associated with 90-day mortality (Table 2). On a clinically interpretable scale, the odds ratio was 1.94 (95% confidence interval 1.26–2.99; *p* = 0.003) per 10 years of age, 1.58 (1.19–2.10; *p* = 0.002) per 5 NIHSS points, and 1.05 (1.00–1.11; *p* = 0.040) per 10 mg/dL of glucose. Admission CAR was not independently associated with mortality after adjustment (odds ratio 1.46 per 1-SD increase in log CAR, 95% confidence interval 0.91–2.33; *p* = 0.115; corresponding per-log-unit odds ratio 1.28; Table 2).

Adding admission CAR to the clinical reference model changed the apparent area under the receiver operating characteristic curve from 0.768 (95% confidence interval 0.676–0.860) to 0.783 (0.693–0.874); the paired difference was +0.016 (95% confidence interval −0.024 to +0.055; DeLong *p* = 0.444), and the likelihood-ratio test was not significant (*p* = 0.113; Table 3). After bootstrap optimism correction, the two point estimates were close (0.750 for the clinical reference model versus 0.754 for the CAR-augmented model; nested-bootstrap 95% confidence intervals 0.662 to 0.858 and 0.674 to 0.867, respectively). These intervals are marginal intervals for each model separately; their overlap is not a test of the difference between the models, and the formal comparison is the paired DeLong test and likelihood-ratio test reported above. The Brier score was 0.139 for the clinical reference model and 0.136 for the CAR-augmented model (optimism-corrected 0.150 and 0.151, respectively), the calibration slope was 0.874 and 0.821, respectively, and the Akaike information criterion changed from 130.0 to 129.5; apparent and optimism-corrected calibration and overall model-fit metrics are reported in full in Supplementary Table S2. Net reclassification improvement (+0.204, 95% confidence interval −0.150 to +0.682) and integrated discrimination improvement (+0.015, 95% confidence interval −0.001 to +0.112) both included the null value and are reported in Supplementary Table S3 only. In the exploratory LASSO check, all four candidate variables, including log admission CAR, were retained at both the minimum-deviance penalty and the one-standard-error penalty; log admission CAR carried the smallest standardized coefficient (+0.053 at the one-standard-error penalty, compared with +0.10 to +0.41 for the clinical predictors) and was the first variable to be eliminated as the penalty increased further. This is consistent with, but does not by itself establish, limited independent information from CAR; penalized selection is unstable at this event count and the result is reported for completeness only.

### 3.3. Follow-Up and Change-in-CAR Analyses

Follow-up (intensive care) CAR did not add incremental value to the clinical reference model (*n* = 142; 31 events): the area under the curve changed from 0.770 (95% confidence interval 0.678–0.861) to 0.777 (0.688–0.866) (ΔAUC +0.008, 95% confidence interval −0.027 to +0.042; DeLong *p* = 0.665; likelihood-ratio *p* = 0.159; optimism-corrected area under the curve 0.747). Change in CAR did not improve model performance (ΔAUC +0.001, 95% confidence interval −0.002 to +0.004; likelihood-ratio *p* = 0.945; odds ratio 1.00 per unit). In a nested analysis restricted to patients with both measurements (*n* = 141; 31 events), adding follow-up CAR to a model that already contained admission CAR did not improve discrimination (ΔAUC +0.000, 95% confidence interval −0.005 to +0.005; DeLong *p* = 0.911; likelihood-ratio *p* = 0.822; odds ratio 1.06, 95% confidence interval 0.63–1.79) and worsened the Akaike information criterion (129.5 to 131.4); change in CAR behaved identically (likelihood-ratio *p* = 0.956; Supplementary Table S4). Restricting the follow-up sample to CAR measured within 24 h (*n* = 116; 27 events; ΔAUC +0.021, 95% confidence interval −0.030 to +0.072; likelihood-ratio *p* = 0.080) or within 48 h (*n* = 122; 28 events; ΔAUC +0.016, 95% confidence interval −0.023 to +0.055; likelihood-ratio *p* = 0.169) of admission did not change this pattern. The primary admission CAR analysis was insensitive to the assumed C-reactive protein detection limit, with likelihood-ratio *p*-values from 0.090 to 0.113 across detection limits of 1, 3, and 5 mg/L (Supplementary Table S5). To account for the variable interval between measurements, the rate of change in CAR (per hour) was modeled and likewise did not improve prediction (LR *p* = 0.801; Supplementary Table S6).

**Figure 2:**
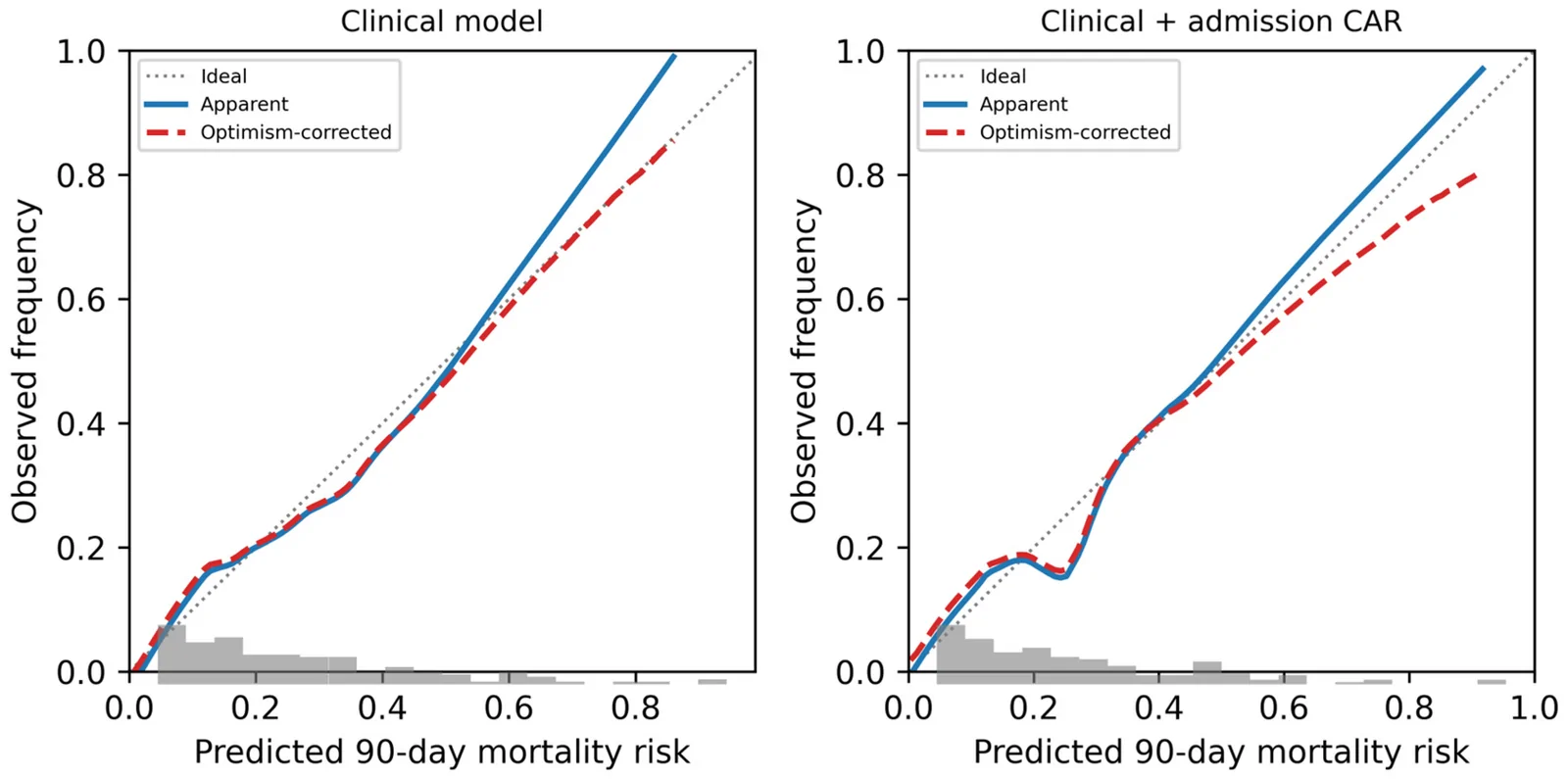
Calibration of the clinical prediction model with and without admission CAR. Apparent calibration (locally weighted scatterplot smoother of observed 90-day mortality versus predicted risk) is shown together with the bootstrap optimism-corrected calibration curve (1000 resamples). Optimism was estimated by Harrell’s procedure: in each resample, the model was refitted, optimism was taken as the difference between that refitted model’s calibration curve in its own resample and the calibration curve of the same refitted model applied to the original data, and the mean of these differences was subtracted from the apparent curve. Mean optimism was +0.021 for the clinical model and +0.033 for the model augmented with admission CAR; the dotted line denotes ideal calibration, and the gray histogram beneath each panel shows the distribution of predicted risk. **Left**, clinical model (age, admission NIHSS score, admission glucose); **right**, clinical model augmented with admission CAR.

Calibration of the clinical reference model and the clinical reference model augmented with admission CAR was similar; apparent and bootstrap optimism-corrected calibration curves, with the distribution of predicted risk, are shown in Figure 2.

### 3.4. Functional Outcome and Sensitivity Analyses

Admission CAR was not associated with poor 90-day functional outcome after adjustment for age, admission NIHSS score, and admission glucose (odds ratio 1.09, 95% confidence interval 0.83–1.44; *p* = 0.530; complete-case *n* = 139; Table 4).

In a clinical-course sensitivity model additionally adjusting for sepsis during hospitalization or intensive care, CAR remained non-significant. Because sepsis arose as a post-admission clinical-course variable and was not uniformly defined by Sepsis-3 criteria, its association estimate was imprecise and is reported in the Supplementary Materials (Table S7) rather than in the primary analysis.

There was no significant interaction between admission CAR and NIHSS score. NIHSS-stratified analyses showed no statistically significant CAR association within mild, moderate, or severe neurological severity categories (Table 4).

Because stroke-specific variables were absent from the primary reference model, the incremental analysis was repeated after expanding the reference model with the recorded variables available for sensitivity analysis: Glasgow Coma Scale score (available for all 146 patients), receipt of reperfusion treatment (intravenous thrombolysis or mechanical thrombectomy, determinable for 144), and time from symptom onset (recorded for 121). Adding Glasgow Coma Scale score raised the reference discrimination from 0.768 to 0.785 and reduced the incremental contribution of admission CAR to ΔAUC by +0.007 (95% confidence interval −0.029 to +0.044; DeLong *p* = 0.693; likelihood-ratio *p* = 0.154). Adding reperfusion treatment gave ΔAUC +0.010 (−0.032 to +0.053; DeLong *p* = 0.632; likelihood-ratio *p* = 0.103); mechanical thrombectomy status was not recorded for two patients, and neither received intravenous thrombolysis, so their reperfusion status could not be determined, and every model containing the reperfusion term is fitted in *n* = 139 (31 events). With both variables in the reference model (*n* = 139; 31 events), the incremental contribution fell to ΔAUC +0.002 (−0.035 to +0.039; DeLong *p* = 0.913; likelihood-ratio *p* = 0.133), and with time from symptom onset additionally included (*n* = 115; 24 events) it was −0.002 (−0.026 to +0.021; DeLong *p* = 0.850; likelihood-ratio *p* = 0.474). The incremental contribution of CAR therefore did not increase when the reference model was made more complete; in every expansion tested, it was smaller than the +0.016 of the primary comparison and lay close to zero (Supplementary Table S8). Infarct volume, premorbid modified Rankin Scale score, and TOAST subtype were not available in the source dataset and could not be examined.

## 4. Discussion

In this single-center cohort of critically ill patients with acute ischemic stroke, admission CAR was not independently associated with 90-day mortality after adjustment for age, admission NIHSS score, and admission glucose (odds ratio 1.46 per 1-SD log CAR, 95% confidence interval 0.91–2.33), and adding CAR to the clinical reference model produced a small and non-significant change in discrimination (ΔAUC +0.016, 95% confidence interval −0.024 to +0.055; DeLong *p* = 0.444; likelihood-ratio *p* = 0.113). The optimism-corrected point estimates were close (0.750 versus 0.754), but the model-specific nested-bootstrap confidence intervals are wide (0.662 to 0.858 and 0.674 to 0.867), and this proximity is not evidence of equivalence; neither follow-up CAR nor change in CAR improved prediction. These results indicate limited incremental prognostic value for CAR in this setting. The wide confidence intervals around the incremental estimates mean that absence of evidence here should not be read as evidence of no effect; any independent contribution of CAR beyond established clinical predictors remains imprecisely estimated and was not measurable in this sample.

The size of the cohort constrains what can be concluded, and that constraint is better stated quantitatively than qualitatively. In a simulation conditioned on the observed admission cohort (*n* = 141; 31 events) with the clinical reference model held fixed (5000 replications; α = 0.05), the smallest incremental effect this design could detect with 80% power was an odds ratio of approximately 1.89 per 1-SD increase in log CAR, evaluated with the likelihood-ratio test used for the primary comparison. Under the null, the simulation returned a rejection rate of 0.050, confirming correct calibration. This quantity is a property of the design—sample size, event count, and the fixed reference model—and was computed without reference to the effect actually observed; it is a retrospective design-sensitivity analysis rather than an observed-power calculation (Supplementary Table S9). Measurable incremental value was not detected; however, limited precision and low power mean that modest or potentially clinically relevant effects cannot be excluded. The width of the confidence interval should be read as a limitation of precision rather than as positive evidence of equivalence. What these data do not support is the stronger claim, common in the biomarker literature, that CAR is an established clinically useful addition to routine predictors in this setting. We make no assumption about how large an effect would have to be in order to be clinically useful; we report only that no incremental value was measurable at this sample size.

A second constraint on interpretation is multiplicity. This paper reports a family of fourteen tests of incremental value: the prespecified primary comparison together with thirteen further tests across the follow-up, change-based, time-window, detection-limit, nested, and expanded-reference-model analyses. None was adjusted for multiple comparisons. The smallest *p*-value anywhere in that family is 0.080, for follow-up CAR restricted to samples drawn within 24 h. A Bonferroni threshold for fourteen tests would be 0.0036, roughly twenty-two times smaller than that value; after Holm adjustment no test reaches an adjusted *p* below 1.000, and after Benjamini–Hochberg control of the false discovery rate, the smallest adjusted value is 0.263 (Supplementary Table S10). This matters because several unadjusted values in the 0.080 to 0.113 range sit close enough to conventional thresholds to invite reading as a trend towards significance. That reading is not available here: none remained significant under any of the three multiplicity corrections evaluated (Bonferroni, Holm, and Benjamini–Hochberg), and multiplicity acts in the direction of producing such near-threshold values by chance rather than against it. We therefore treat every analysis other than the prespecified primary comparison as hypothesis-generating.

This pattern can be reconciled with earlier reports that linked CAR to outcome [23,27]. Those analyses primarily quantified the association between CAR and mortality or functional outcome rather than its added value over a model already containing the NIHSS score, age, and glucose. Because CAR reflects systemic inflammation and nutritional reserve, much of its prognostic signal may overlap with the same neurological severity, age, metabolic stress, and intensive care complications that the clinical reference model already captures, leaving little independent information once those variables are present. The cohort studied here was restricted to critically ill patients, in whom inflammatory and severity markers are concentrated and correlated, which may further reduce the marginal information carried by CAR. These observations describe statistical overlap and do not support any causal interpretation of CAR with respect to outcome.

The restriction of this cohort to intensive care patients deserves explicit treatment, because it bears directly on how far the result can be generalized. Selection into intensive care conditions the cohort on severity: patients enter precisely because their neurological deficit, level of consciousness, or systemic physiology is already abnormal, so the severity signal that an inflammatory marker would otherwise carry is concentrated, mutually correlated, and largely captured by the variables already in the model. Restriction of the range of severity in this way is expected to attenuate the discrimination an additional marker can contribute; conditioning on admission to intensive care is at the same time a selection step, and selection effects of this kind may operate in an uncertain direction. Neither can be demonstrated directly in a cohort that contains no unselected comparison group, so we do not assert a net direction. The estimate should therefore be read as specific to this population, which is also the population in which CAR has most often been proposed as a triage or risk-stratification aid. The contrast with larger unselected cohorts is instructive. In a stroke registry of 1484 patients, CAR was independently associated with 90-day functional outcome and added measurable reclassification value to a clinical model (net reclassification improvement 0.148, 95% confidence interval 0.043 to 0.254) [38], and in a cohort of 1595 patients CAR predicted stroke-associated pneumonia with good discrimination [39], that is, it performed well where the outcome is an infectious complication and where the population still contained substantial variation in severity. In the critically ill subset studied here, where much of that variation has already been selected away, no incremental value was detectable. Taken together, these observations are consistent with the hypothesis that the incremental performance of CAR may differ across severity and case-mix distributions rather than being a fixed property of the marker; the present data cannot establish that relationship, only that no incremental value was measurable in this severely ill population. Our result should therefore not be generalized to all-comer stroke populations, and we do not do so.

It is useful to separate three properties that the biomarker literature frequently conflates. A prognostic biomarker is one whose values differ between patients who do and do not experience the outcome; CAR clearly satisfies this in unselected stroke populations, and our unadjusted data are not inconsistent with it. A severity marker is one whose values track the same underlying illness burden that clinicians already measure by other means; the correlation of CAR with neurological severity, admission hyperglycemia, and subsequent intensive care complications in this cohort is consistent with that role. An incrementally useful predictor is one that changes the predicted risk after the routinely available predictors are already in the model, and only this third property justifies measuring a marker for prognostic purposes at the bedside. Our results are compatible with CAR possessing the first two properties and do not support the third in critically ill patients with acute ischemic stroke. For the clinician, the practical translation is that in this cohort, a high CAR may largely reflect information already captured by established severity measures—the NIHSS score, the admission glucose, and the patient’s age—rather than clearly providing additional prognostic information; however, a modest independent contribution cannot be excluded.

Serial measurement showed the same pattern. Although repeated inflammatory biomarkers are attractive in intensive care, follow-up CAR and change in CAR did not improve prediction beyond a simple clinical model. One explanation is that follow-up CAR is influenced by complications, treatment intensity, sampling time, nutrition, hydration, infection, hepatic synthesis, and the critical illness course. Without standardized sampling intervals, follow-up CAR should be interpreted cautiously. Because the timing of the second sample was not standardized, these analyses test whether a second CAR measurement carries incremental prognostic information at an early intensive care time point; they do not constitute an evaluation of CAR kinetics, and no claim is made about the rate or trajectory of change. The time-normalized rate analysis and the restriction to samples drawn within 24 and 48 h were performed for this reason and did not alter the conclusion, but they mitigate rather than remove the limitation.

Sepsis was deliberately handled as a clinical-course variable rather than as a baseline predictor. The available dataset records sepsis as a documented hospitalization or intensive care complication, but it does not contain sufficient information for uniform retrospective Sepsis-3 adjudication. Treating sepsis as a baseline independent predictor could therefore create temporal ambiguity and overadjustment. The dataset carries no reliable onset timestamp for sepsis, so its position in time relative to the admission and early intensive care blood samples that define admission and follow-up CAR cannot be established, and the proportion of patients in whom sepsis was recognized before rather than after the two samples were drawn cannot be determined from these records. Adjusting a prognostic model for a variable that plausibly lies on the causal pathway between systemic inflammation and death, and whose timing relative to the exposure is unknown, would constitute overadjustment and would yield an uninterpretable estimate. For this reason, sepsis was moved out of the primary prognostic model and retained only as a clinical-course sensitivity variable. That analysis is reported for the single purpose of documenting that the qualitative conclusion for CAR is unchanged after the adjustment: intensive care CAR remained non-significant (odds ratio 1.19, 95% confidence interval 0.80–1.77; *p* = 0.401). The magnitude of the sepsis estimate itself should not be interpreted. Sepsis was documented in 26 of the 31 deaths and in 10 of the 111 survivors within this model cohort, so the data approach quasi-complete separation; re-estimation with Firth bias-reduced logistic regression attenuated the odds ratio from 37.13 to 27.44 (95% profile penalized-likelihood confidence interval 9.39–93.55) while leaving it large and imprecise, which indicates that the point estimate is numerically unstable under near-separation and should not be read as an effect size.

The inclusion of 90-day modified Rankin Scale data also clarifies the clinical relevance of CAR. Although mortality is important, acute ischemic stroke studies usually require functional interpretation. Admission CAR was not associated with poor functional outcome after adjustment for age, NIHSS, and glucose, suggesting that any mortality signal is not clearly translated into broader disability prediction.

This study has limitations. It is retrospective and single-center, and the cohort represents intensive care patients rather than all patients with acute ischemic stroke. The sample size and number of deaths limit model complexity. Sepsis could not be adjudicated using uniform Sepsis-3 criteria. Intensive care laboratory sampling times were not standardized. Important stroke predictors, including infarct volume, premorbid modified Rankin Scale, TOAST classification, and onset-to-treatment metrics, were not uniformly available. These limitations mean that the results require external validation in larger multicenter cohorts. Finally, and most importantly for interpretation, the model presented here should be understood as an analytic reference against which the incremental value of a biomarker was tested, and not as a deployable risk-prediction tool. It was not developed, calibrated, or validated for clinical use; no risk threshold or decision rule is proposed; and no external validation was performed. The bootstrap optimism-corrected performance reported above describes internal validity within this dataset only and must not be read as an estimate of performance in a new population or setting.

## 5. Conclusions

In critically ill patients with acute ischemic stroke, admission CAR was not independently associated with 90-day mortality and did not demonstrate measurable incremental prognostic value in this cohort beyond age, neurological severity, and admission glucose; follow-up and change-based CAR likewise did not improve prediction. CAR should be interpreted as a complementary marker of inflammatory burden rather than a stand-alone prognostic tool; its incremental contribution in this setting remains imprecisely estimated and was not measurable at this sample size. Because the second CAR measurement was not obtained at a standardized time, these analyses test incremental prognostic information at an early intensive care time point rather than constituting a full evaluation of CAR kinetics. The clinical model used here is an analytic reference rather than a deployable prediction tool, and these conclusions derive from a single-center cohort without external validation; confirmation in larger, prospectively sampled, multicenter populations is required before this negative finding is generalized to less severely ill stroke populations.

## Figures and Tables

**Table 1:** Baseline characteristics according to 90-day mortality.

Variable	Overall	Survivors	Non-Survivors	*p*-Value
Age, years	70.0 (61.0–80.0)	69.0 (59.5–77.8)	78.5 (68.0–84.5)	0.002
Male sex	68/146 (46.6)	55/114 (48.2)	13/32 (40.6)	0.445
Hypertension	108/144 (75.0)	87/112 (77.7)	21/32 (65.6)	0.165
Diabetes mellitus	40/144 (27.8)	32/112 (28.6)	8/32 (25.0)	0.691
Atrial fibrillation	34/144 (23.6)	24/112 (21.4)	10/32 (31.2)	0.249
Admission NIHSS	9.0 (4.0–15.0)	7.0 (4.0–12.0)	14.0 (9.8–18.5)	<0.001
Admission GCS	14.0 (10.2–15.0)	14.0 (11.0–15.0)	11.0 (7.8–13.0)	<0.001
Admission glucose, mg/dL	129.0 (108.5–172.0)	124.5 (105.8–166.8)	157.0 (122.5–222.5)	0.007
Admission CAR	1.4 (0.6–4.7)	1.3 (0.5–4.6)	2.1 (0.7–14.3)	0.155
ICU CAR	2.1 (0.8–6.6)	2.0 (0.8–5.5)	3.2 (0.9–11.7)	0.093
Delta CAR	0.1 (0.0–0.5)	0.1 (0.0–0.5)	0.2 (0.0–2.2)	0.540
IV thrombolysis	44/145 (30.3)	38/113 (33.6)	6/32 (18.8)	0.106
Mechanical thrombectomy	40/144 (27.8)	28/112 (25.0)	12/32 (37.5)	0.164
Sepsis during hospitalization/ICU	37/146 (25.3)	10/114 (8.8)	27/32 (84.4)	<0.001
Intubation	47/146 (32.2)	16/114 (14.0)	31/32 (96.9)	<0.001
Vasopressor requirement	40/146 (27.4)	9/114 (7.9)	31/32 (96.9)	<0.001
90-day mRS	4.0 (2.0–5.0)	3.0 (2.0–4.0)	6.0 (6.0–6.0)	<0.001

Note: Continuous values are median (interquartile range); categorical values are *n*/N (%). CAR, C-reactive protein-to-albumin ratio; GCS, Glasgow Coma Scale; ICU, intensive care unit; NIHSS, National Institutes of Health Stroke Scale.

**Table 2:** Primary logistic regression models for 90-day mortality.

Variable	Scale	OR (95% CI)	*p*-Value
Age	per 10 years	1.94 (1.26–2.99)	0.003
Admission NIHSS	per 5 points	1.58 (1.19–2.10)	0.002
Admission glucose	per 10 mg/dL	1.05 (1.00–1.11)	0.040
Admission CAR	per 1-SD log CAR	1.46 (0.91–2.33)	0.115

Note: Estimates are from the primary multivariable model (*n* = 141; 31 events). CAR was analyzed as log(CAR); the SD of log(CAR) was 1.536 (per-log-unit odds ratio 1.28). CAR, C-reactive protein-to-albumin ratio; CI, confidence interval; NIHSS, National Institutes of Health Stroke Scale; OR, odds ratio.

**Table 3:** Incremental discrimination of CAR variables added to the clinical reference model.

Model	Apparent AUC (95% CI)	Optimism-Corrected AUC (95% CI)	AUC Difference vs Clinical (95% CI)	DeLong *p*	LR *p*
Clinical reference (admission cohort, *n* = 141)	0.768 (0.676–0.860)	0.750 (0.662–0.858)	reference	—	—
+ Admission CAR	0.783 (0.693–0.874)	0.754 (0.674–0.867)	+0.016 (−0.024 to +0.055)	0.444	0.113
Clinical reference (follow-up cohort, *n* = 142)	0.770 (0.678–0.861)	0.752 (0.671–0.854)	reference	—	—
+ Follow-up CAR	0.777 (0.688–0.866)	0.747 (0.674–0.867)	+0.008 (−0.027 to +0.042)	0.665	0.159

Note: The clinical reference model included age, admission NIHSS score, and admission glucose. Admission CAR and follow-up CAR analyses were performed in separate complete-case cohorts (*n* = 141 and *n* = 142, respectively), each with its own clinical reference model; the ΔAUC for each CAR variable is relative to the clinical reference within the same cohort. CAR was analyzed as log(CAR); change in CAR was analyzed on its raw scale and is reported in the text (ΔAUC +0.001; likelihood-ratio *p* = 0.945). Optimism-corrected metrics are from 1000 bootstrap resamples, and the confidence interval for each optimism-corrected AUC is from a nested bootstrap (600 outer resamples, each with 120 inner resamples). The AUC difference column is the paired difference in apparent AUC between the augmented and the reference model within the same cohort, denoted ΔAUC elsewhere in the text. Brier scores, calibration slope and intercept, and the Akaike information criterion for the same four models are reported separately in Supplementary Table S2, so that this table presents discrimination alone. AUC, area under the receiver operating characteristic curve; CAR, C-reactive protein-to-albumin ratio; CI, confidence interval; LR, likelihood-ratio.

**Table 4:** Secondary and sensitivity analyses.

Analysis	Effect Tested	*n*/Events	OR (95% CI)	*p*-Value
Secondary outcome: poor functional outcome (90-day mRS 3–6)	Admission CAR, log(CAR)	139/93	1.09 (0.83–1.44)	0.530
Effect modification: admission CAR × NIHSS interaction	Admission CAR × NIHSS	141/31	0.98 (0.95–1.01)	0.236
Subgroup: NIHSS stratum 0–4	Admission CAR, log(CAR)	37/4	1.41 (0.75–2.64)	0.291
Subgroup: NIHSS stratum 5–14	Admission CAR, log(CAR)	67/13	1.42 (0.97–2.09)	0.071
Subgroup: NIHSS stratum ≥ 15	Admission CAR, log(CAR)	37/14	0.95 (0.56–1.60)	0.850

Note: Poor functional outcome was defined as 90-day modified Rankin Scale score 3–6. NIHSS strata were 0–4, 5–14, and ≥15. CAR terms were analyzed as log(CAR), and the odds ratios in this table are expressed per one natural-log unit of CAR; the primary admission CAR estimate reported in the Abstract and in Table 2 is expressed per 1-SD increase in log(CAR) (1 SD = 1.536 log units), so the two scales are not directly comparable. The interaction term is the change in the per-log-unit CAR odds ratio per one-point increase in the admission NIHSS score. NIHSS-stratified estimates are within-stratum univariable owing to limited events per stratum. Rows are grouped by analysis type: one secondary outcome, one test of effect modification, and three prespecified severity subgroups.

## Data Availability

The datasets used and analyzed during the current study are available from the corresponding author upon reasonable request. An earlier version of this work was posted as a preprint on Research Square (DOI: 10.21203/rs.3.rs-9063890/v1).

## Supplementary Materials

The following supporting information can be downloaded at: https://www.mdpi.com/article/10.3390/diagnostics16162578/s1: Table S1: Per-variable missing data for the variables used in the principal CAR models (base N = 146); Table S2: Calibration and overall model fit for the models in Table 3; Table S3: Exploratory reclassification metrics for admission CAR added to the clinical reference model; Table S4: Nested serial analysis: incremental value of follow-up CAR and change in CAR conditional on admission CAR; Table S5: Sensitivity of the primary admission-CAR incremental analysis to the assumed CRP detection limit (CRP = 0 imputed to DL/√2); Table S6: Time-normalized change-in-CAR (rate per hour) sensitivity analysis for 90-day mortality; Table S7: Clinical-course sensitivity analysis: association of sepsis during hospitalization or intensive care, and intensive care CAR adjusted for clinical course, with 90-day mortality; Table S8: Sensitivity analysis: incremental value of admission CAR when the clinical reference model is expanded with additional stroke-specific variables; Table S9: Design sensitivity: simulated power to detect an incremental effect of admission CAR beyond the clinical reference model; Table S10: Multiplicity: unadjusted and adjusted *p*-values for the family of incremental-value tests.

## Abbreviations

AIC, Akaike information criterion; AUC, area under the receiver operating characteristic curve; ΔAUC, difference in the area under the receiver operating characteristic curve between two nested models; CAR, C-reactive protein-to-albumin ratio; CI, confidence interval; CRP, C-reactive protein; GCS, Glasgow Coma Scale; ICU, intensive care unit; LR, likelihood-ratio; mRS, modified Rankin Scale; NIHSS, National Institutes of Health Stroke Scale; OR, odds ratio.
