# Supplementary material for: Incremental Prognostic Value of the C-Reactive Protein-to-Albumin Ratio Beyond a Parsimonious Clinical Reference Model in Critically Ill Patients with Acute Ischemic Stroke
Source: Diagnostics (Basel). 2026 Aug 15;16(16):2578. doi: 10.3390/diagnostics16162578 (PMC13512708; doi:10.3390/diagnostics16162578)
Supplement: Supplementary file 1 [file diagnostics-16-02578-s001.zip › diagnostics-4440658-supplementary.pdf]

Supplementary Materials

Incremental Prognostic Value of the C-Reactive Protein-to-Albumin Ratio Beyond a Parsimonious Clinical Reference Model in Critically Ill Patients with Acute Ischemic Stroke

Table S1. Per-variable missing data for the variables used in the principal CAR models (base N = 146).

Variable	Available, n	Missing, n (%)
**Age**	146	0 (0.0%)
**Admission NIHSS**	145	1 (0.7%)
**Admission glucose**	143	3 (2.1%)
**Emergency department CRP**	144	2 (1.4%)
**Intensive care CRP**	146	0 (0.0%)
**Admission serum albumin**	146	0 (0.0%)
**Intensive care serum albumin**	146	0 (0.0%)
**Admission CAR, log(CAR)**	144	2 (1.4%)
**Intensive care CAR, log(CAR)**	146	0 (0.0%)
**90-day mortality**	146	0 (0.0%)

Note: *Each model used complete cases for its variables. Admission CAR uses admission CRP and admission serum albumin; intensive care CAR uses the corresponding intensive care values. CAR, C-reactive protein-to-albumin ratio; CRP, C-reactive protein; NIHSS, National Institutes of Health Stroke Scale.*

Table S2. Calibration and overall model fit for the models in Table 3.

Model	Apparent Brier	Corrected Brier	Calibration slope (95% CI)	Calibration intercept	AIC
**Clinical reference (admission cohort, n = 141)**	0.139	0.150	0.874 (0.519–1.357)	0.000	130.0
**+ Admission CAR**	0.136	0.151	0.821 (0.475–1.277)	0.008	129.5
**Clinical reference (follow-up cohort, n = 142)**	0.138	0.149	0.872 (0.502–1.348)	0.004	130.1
**+ Follow-up CAR**	0.137	0.151	0.819 (0.456–1.249)	0.010	130.1

Note: *These metrics were previously reported as additional columns of main-text Table 3 and were moved here so that Table 3 presents discrimination alone. Values are unchanged. Optimism-corrected estimates are from 1000 bootstrap resamples. AIC, Akaike information criterion; CAR, C-reactive protein-to-albumin ratio; CI, confidence interval.*

Table S3. Exploratory reclassification metrics for admission CAR added to the clinical reference model.

Metric	Estimate (95% CI)
**Continuous net reclassification improvement (NRI)**	+0.204 (−0.150 to +0.682)
**Integrated discrimination improvement (IDI)**	+0.015 (−0.001 to +0.112)

Note: *Both estimates included the null value and are reported as exploratory; they are unstable at the available event count and were not used as primary evidence. Decision-curve analysis and restricted cubic spline modeling were not performed because the event count was insufficient to support them. Complete-case sample n = 141; 31 events. CAR, C-reactive protein-to-albumin ratio; CI, confidence interval.*

Table S4. Nested serial analysis: incremental value of follow-up CAR and change in CAR conditional on admission CAR.

Model	Apparent AUC (95% CI)	ΔAUC vs model A (95% CI)	DeLong p	LR p	Added-term OR (95% CI)	AIC
**A: clinical reference + log admission CAR**	0.783 (0.693–0.874)	reference	—	—	—	129.5
**B: A + log follow-up CAR**	0.784 (0.693–0.874)	+0.000 (−0.005 to +0.005)	0.911	0.822	1.06 (0.63–1.79)	131.4
**B′: A + change in CAR (raw)**	0.784 (0.694–0.875)	+0.001 (−0.001 to +0.004)	0.343	0.956	1.00 (0.95–1.05)	131.5

Note: *Common complete-case cohort with both CAR measurements: n = 141; 31 events. This analysis addresses whether a second CAR measurement carries information once the admission value is already in the model. Neither follow-up CAR nor change in CAR improved discrimination, and both worsened the Akaike information criterion. AIC, Akaike information criterion; AUC, area under the receiver operating characteristic curve; CAR, C-reactive protein-to-albumin ratio; CI, confidence interval; LR, likelihood-ratio; OR, odds ratio.*

Table S5. Sensitivity of the primary admission-CAR incremental analysis to the assumed CRP detection limit (CRP = 0 imputed to DL/√2).

Detection limit (mg/L)	ΔAUC vs clinical (95% CI)	LR p	CAR OR (per log unit)
**1**	+0.016 (−0.024 to +0.055)	0.113	1.28
**3**	+0.016 (−0.027 to +0.058)	0.096	1.30
**5**	+0.017 (−0.028 to +0.061)	0.090	1.31

Note: *The primary result was insensitive to the assumed detection limit (LR p 0.090–0.113 across DL 1, 3, and 5 mg/L). Primary complete-case sample n = 141; 31 events. AUC, area under the receiver operating characteristic curve; CAR, C-reactive protein-to-albumin ratio; CI, confidence interval; DL, detection limit; LR, likelihood-ratio; OR, odds ratio.*

Table S6. Time-normalized change-in-CAR (rate per hour) sensitivity analysis for 90-day mortality.

Model	Apparent AUC (95% CI)	ΔAUC vs clinical (95% CI)	DeLong p	LR p	Rate OR (per unit/h)
**Clinical reference**	0.753 (0.652–0.855)	reference	—	—	—
**+ Change-in-CAR rate**	0.753 (0.650–0.855)	−0.001 (−0.008 to +0.006)	0.832	0.801	0.94 (0.55–1.59)

Note: *Model n = 122; 28 events. The rate of change in CAR was defined as (intensive care CAR − admission CAR) divided by the interval in hours between the two laboratory samples (valid interval n = 125; median 8.7 h). An interval was treated as evaluable only if it fell inside the predefined analytic window of more than 0 h and at most 168 h (7 days); intervals that were negative, exactly zero (both samples carrying the same recorded timestamp), or greater than 168 h were set to missing. The same rule was applied to every interval-dependent analysis in this paper. This analysis addresses the variable measurement interval as a robustness check and is not intended as a claim about biomarker kinetics or velocity. CAR, C-reactive protein-to-albumin ratio; CI, confidence interval; LR, likelihood-ratio; OR, odds ratio.*

Table S7. Clinical-course sensitivity analysis: association of sepsis during hospitalization or intensive care, and intensive care CAR adjusted for clinical course, with 90-day mortality.

Effect tested	n/events	OR (95% CI)	p-value
**Sepsis during hospitalization/ICU**	142/31	37.13 (10.89–126.64)	<0.001
**Intensive care CAR, log(CAR)**	142/31	1.19 (0.80–1.77)	0.401

Note: *Clinical-course model adjusted for age, admission NIHSS score, admission glucose, sepsis during hospitalization or intensive care, and intensive care CAR (n = 142; 31 events). Sepsis arose as a post-admission clinical-course variable without a reliable onset timestamp and was not uniformly defined by Sepsis-3 criteria, so it is reported here rather than in the primary analysis. Sepsis was documented in 26 of the 31 deaths and in 10 of the 111 survivors in this cohort, so the data approach quasi-complete separation and the sepsis odds ratio must not be interpreted quantitatively. Re-estimation with Firth bias-reduced logistic regression attenuated the sepsis odds ratio from 37.13 to 27.44 (95% profile penalized-likelihood confidence interval 9.39–93.55) while leaving it large and imprecise; the point estimate is numerically unstable under near-separation and should not be read as an effect size. Intensive care CAR remained non-significant under both estimators (maximum likelihood 1.19, 0.80–1.77; Firth 1.17, 0.80–1.73). CAR, C-reactive protein-to-albumin ratio; CI, confidence interval; ICU, intensive care unit; NIHSS, National Institutes of Health Stroke Scale; OR, odds ratio.*

Table S8. Sensitivity analysis: incremental value of admission CAR when the clinical reference model is expanded with additional stroke-specific variables.

Reference model	n / events	Reference AUC (95% CI)	With admission CAR AUC (95% CI)	ΔAUC (95% CI)	DeLong p	LR p
**Clinical reference (age, NIHSS, glucose) — primary**	141 / 31	0.768 (0.676–0.860)	0.783 (0.693–0.874)	+0.016 (−0.024 to +0.055)	0.444	0.113
**+ Glasgow Coma Scale score**	141 / 31	0.785 (0.698–0.872)	0.792 (0.705–0.879)	+0.007 (−0.029 to +0.044)	0.693	0.154
**+ Reperfusion treatment**	139 / 31	0.766 (0.674–0.859)	0.777 (0.685–0.869)	+0.010 (−0.032 to +0.053)	0.632	0.103
**+ Glasgow Coma Scale score + reperfusion treatment**	139 / 31	0.783 (0.696–0.869)	0.785 (0.696–0.873)	+0.002 (−0.035 to +0.039)	0.913	0.133
**+ Glasgow Coma Scale score + reperfusion treatment + onset time**	115 / 24	0.802 (0.712–0.892)	0.799 (0.706–0.893)	−0.002 (−0.026 to +0.021)	0.850	0.474

Note: *Reperfusion treatment was defined as receipt of intravenous thrombolysis or mechanical thrombectomy. Mechanical thrombectomy status was not recorded for two patients, and because neither received intravenous thrombolysis their reperfusion status could not be determined; both are therefore absent from the complete-case samples of the models that contain the reperfusion term, which is why those models have n = 139 rather than 141 and n = 115 rather than 117. Onset time is the recorded interval from symptom onset to presentation and was available for 121 patients. Odds ratios for admission CAR per 1-SD increase in log CAR were 1.46 (0.91–2.33), 1.41 (0.88–2.28), 1.49 (0.92–2.43), 1.46 (0.89–2.39), and 1.23 (0.69–2.20) across the five models respectively; each odds ratio is scaled by the standard deviation of log CAR within that model's own complete-case sample. The events-per-variable ratio falls from 7.8 in the primary model to 3.4 in the fullest model, so the expanded models are reported as sensitivity analyses and not as alternative primary models. Infarct volume, premorbid modified Rankin Scale score, and TOAST subtype were not uniformly recorded and could not be included. AUC, area under the receiver operating characteristic curve; CAR, C-reactive protein-to-albumin ratio; CI, confidence interval; LR, likelihood-ratio; NIHSS, National Institutes of Health Stroke Scale.*

Table S9. Design sensitivity: simulated power to detect an incremental effect of admission CAR beyond the clinical reference model.

Incremental odds ratio per 1-SD increase in log CAR	Simulated power
**1.00 (null; type I error check)**	0.050
**1.20**	0.124
**1.40**	0.318
**1.60**	0.536
**1.80**	0.728
**1.90**	0.810
**2.00**	0.860
**2.20**	0.931
**2.40**	0.968
**2.60**	0.988

Note: *Outcomes were simulated from the fitted clinical reference model in the observed admission cohort (n = 141; 31 events), adding a specified effect of standardized log admission CAR; 5000 replications per odds ratio, two-sided α = 0.05, seed 42. Each simulated dataset was tested with the likelihood-ratio test, the same test used for the primary comparison, so that the simulated rejection rate refers to the test actually reported. The rejection rate of 0.050 under the null confirms correct calibration of the simulation. Linear interpolation across the grid gives a minimum detectable effect of approximately 1.89 at 80% power. This is a design-sensitivity analysis conducted during revision: it describes which effect sizes this design could have detected and is determined by the sample size, the event count, and the fixed reference model rather than by the effect actually observed. The grid was selected independently of the observed estimate and deliberately contains neither the observed odds ratio nor the observed upper confidence limit, because power evaluated at an observed effect estimate is a deterministic function of the observed p value and carries no information beyond it. CAR, C-reactive protein-to-albumin ratio.*

Table S10. Multiplicity: unadjusted and adjusted p-values for the family of incremental-value tests.

Test of incremental value	Unadjusted p	Holm-adjusted p	Benjamini-Hochberg adjusted p
**Follow-up CAR, ≤24 h window**	0.080	1.000	0.263
**CRP detection limit 5 mg/L**	0.090	1.000	0.263
**CRP detection limit 3 mg/L**	0.096	1.000	0.263
**Expanded reference + reperfusion treatment**	0.103	1.000	0.263
**Admission CAR (prespecified primary test)**	0.113	1.000	0.263

Test of incremental value	Unadjusted p	Holm-adjusted p	Benjamini-Hochberg adjusted p
**Expanded reference + GCS + reperfusion**	0.133	1.000	0.263
**Expanded reference + GCS**	0.154	1.000	0.263
**Follow-up CAR**	0.159	1.000	0.263
**Follow-up CAR, ≤48 h window**	0.169	1.000	0.263
**Expanded reference + GCS + reperfusion + onset**	0.474	1.000	0.664
**Time-normalized rate of change in CAR**	0.801	1.000	0.956
**Nested: follow-up CAR after admission CAR**	0.822	1.000	0.956
**Change in CAR**	0.945	1.000	0.956
**Nested: change in CAR after admission CAR**	0.956	1.000	0.956

Note: *The family comprises every test of the incremental value of a CAR variable over a clinical reference model reported in this paper (k = 14), ordered by unadjusted p value. No test is significant at the 0.05 level after Holm adjustment or after Benjamini-Hochberg control of the false discovery rate. The Bonferroni threshold for k = 14 is 0.0036, approximately twenty-two times smaller than the smallest unadjusted p value observed (0.080). The primary analysis was prespecified and is listed here for completeness; all other tests are secondary or exploratory and are interpreted as hypothesis-generating. CAR, C-reactive protein-to-albumin ratio; CRP, C-reactive protein; GCS, Glasgow Coma Scale.*
